# Wilhelm Hofmeister (1824–1877) and the ideas of evolutionary embryonization

**DOI:** 10.3897/compcytogen.18.143444

**Published:** 2024-12-18

**Authors:** Ilya A. Gavrilov-Zimin

**Affiliations:** 1 S.I. Vavilov Institute for the History of Science and Technology, St. Petersburg Branch, Russian Academy of Sciences, Universitetskaya nab. 5, St. Petersburg, Russia St. Petersburg Branch, Russian Academy of Sciences St. Petersburg Russia; 2 Zoological Institute, Russian Academy of Sciences, Universitetskaya nab. 1, St. Petersburg, Russia Zoological Institute, Russian Academy of Sciences St. Petersburg Russia

**Figure 1.**
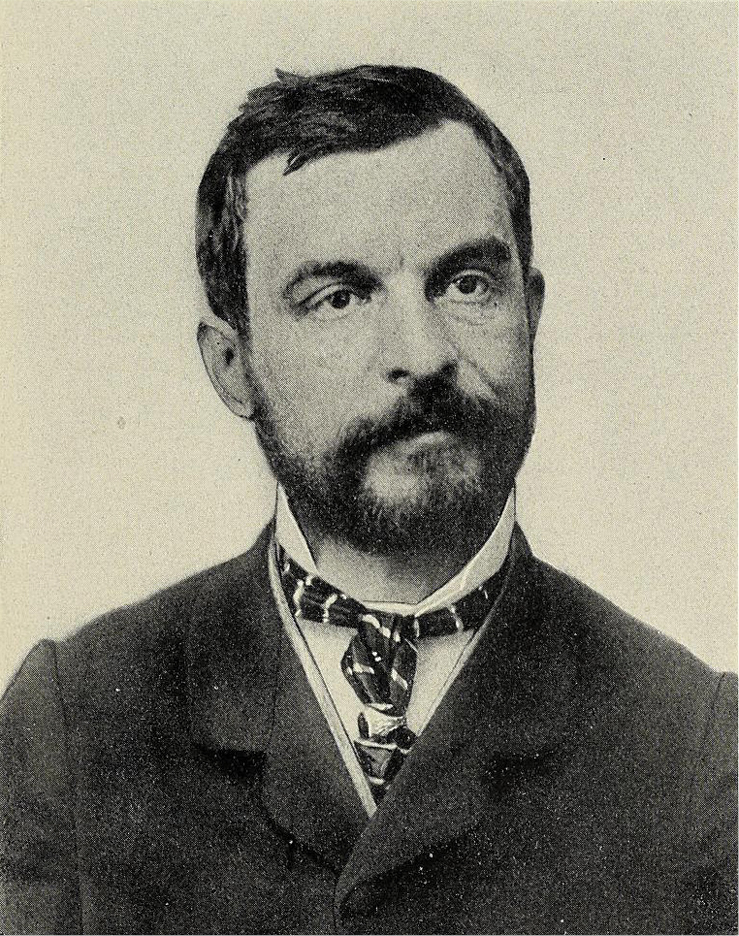
Wilhelm Hofmeister (1824–1877), after Goebel (1905).

This year marks the 200^th^ anniversary of the birth of Friedrich Wilhelm Benedikt Hofmeister, an outstanding German amateur biologist and one of the forerunners of genetics (Fig. 1). Details of his biography have been analyzed in a number of publications (e.g., Goebel 1905; Kaplan and Cooke 1996; Martin 2017, etc.), so there is no need to reproduce them in the present memorial article. In both the educational and scientific literature (see the works cited above), Hofmeister is known primarily as the discoverer of the alternation of generations (gametophyte and sporophyte) in the life cycle of plants. However, this view is not entirely accurate, as formal priority in this matter still belongs to another amateur, the Polish Count Michal Leszczyc-Suminski (1848) (see some details below).

It is also believed (e.g., Kaplan and Cooke 1996: 1650) that Hofmeister’s research strongly influenced another prominent German amateur, Gregor Mendel (1822–1884), and inspired him to conduct the famous experiments in plant hybridization, that laid the foundation for the new biological discipline, genetics.

In the anniversary year, I would like to draw the readers’ attention to the importance of Hofmeister’s work for the development of ideas about the evolutionary embryonization of ontogenesis, which had not previously attracted much attention.

The concepts of evolutionary (or phylogenetic) embryonization (and vs. disembryonization) are now widely used to reconstruct the phylogeny of various groups of plants and animals (see review: Gavrilov-Zimin, 2024). Often, these concepts are so tightly woven into descriptions of the course of evolution of specific taxa that they are not even considered controversial, but are accepted as indisputable facts. But like many other paradigms of modern natural science, these ideas have come a long way from very simple, even naive views that existed long before Darwin’s evolutionary concept, to an increasingly complex and comprehensive understanding of the nature of phenomena. On this long path, the elucidation of the embryonic development of plants has constantly and very much lagged behind embryological studies of animals, despite the fact that objectively plant organisms are much simpler than animals.

If animal embryology and corresponding ideas about embryonization of ontogenesis go back to Aristotle (about 2400 years ago — see modern translations: Aristotle 1940, 1996) and his ideas about the ontogenesis of holometabolous insects, we can speak about plant embryology only since the works of Marcello Malpighi (1628–1694), Nehemiah Grew (1641–1712) and Rudolf Camerer (1665–1721) at the end of the XVII century. Along with the zoologists Antoni Van Leeuwenhoek (1632–1723), Robert Hooke (1635–1703), and Jan Swammerdam (1637–1680), these were the first microscopists, and with their names is associated a major turning point in the history of biology, the boundary between Renaissance biology and the “new” biology. It was then that a huge array of fundamentally new information was discovered, absolutely unknown and not even suspected by biologists of Antiquity and the Renaissance. In Malpighi’ (see, for example, the compilation of his works: Opera omnia, 1687) one can see, in fact, the first scientific botanical illustrations in the history of science, i.e. not only the external appearance of plants, which was depicted by numerous artists of different epochs, but also the subtle internal structure being studied, including the organs of the flower with developing embryos. In the same period of time, the essence of the sexual process in flowering plants, the role of stamens and pistil in the fertilization of the egg cell and formation of the plant embryo was first understood. Apparently, the first to verify this experimentally was the English gardener and amateur botanist Jacob Bobart (1599–1680) and after him the corresponding ideas were developed by the English professional botanist Nehemiah Grew in his book “The Anatomy of Plants” (Grew 1682). Thus, botanical embryology is a child of the early modern era of human history, and the gap between it and animal embryology, which began as early as Aristotle, is as much as 2,000 years. In general, the entire eighteenth century in plant embryology was passed under the sign of constant disputes about preformism and the possibility of sexual reproduction in plants, without any fundamental progress compared to the works of Malpighi and Grew (Baranov 1955). Such progress was only in the 19^th^ century and was associated with the next stage of improvement of microscopic equipment and dissection methods. These innovations made it possible to bring embryological research to a previously unattainable level and to begin a detailed study of the reproductive organs and developing embryos of a wide range of plants, both the most primitive and highly developed. In this regard, we can recall the names of many botanist-embryologists, such as Carl Nägeli (1817–1891), Matthias Schleiden (1804–1881), Eduard Strasburger (1844–1912), Sergei Navashin (1857–1930), and many others.

In the second half of the 19^th^ century, methodological progress was supplemented by a significant conceptual progress associated with the gradual acceptance by more and more biologists of the ideas of Darwinian evolutionism. In the field of embryology, this led first to the realization of the evolutionary variability of animal ontogenesis, and then, as in previous centuries, gradually migrated from zoological embryology to botanical embryology. However, as in the case of animal embryology, the study of plants has historically proceeded in the direction opposite to the course of evolution, that is embryological features of the most complex animals — vertebrates and the most highly developed plants — angiosperms have been studied first and in great detail. Until the middle of the 19^th^ century, all the few studies on the reproductive biology of higher spore plants – mosses, horsetails, club mosses, ferns, and algae – were carried out in the context of attempts to automatically transfer the already formed ideas about the structure of flower, seed, and fruit to something that had nothing homologous with these structures. For example, the spores of these plants were likened to seeds, then to pollen grains, and the sporangia themselves to a flower. There have been cases of the opposite meaning, quite curiosities, in which the spermatozoa of mosses and algae, observed under the microscope, have been thought to be either infusoria or some kind of “monads”.

**Figure 2.**
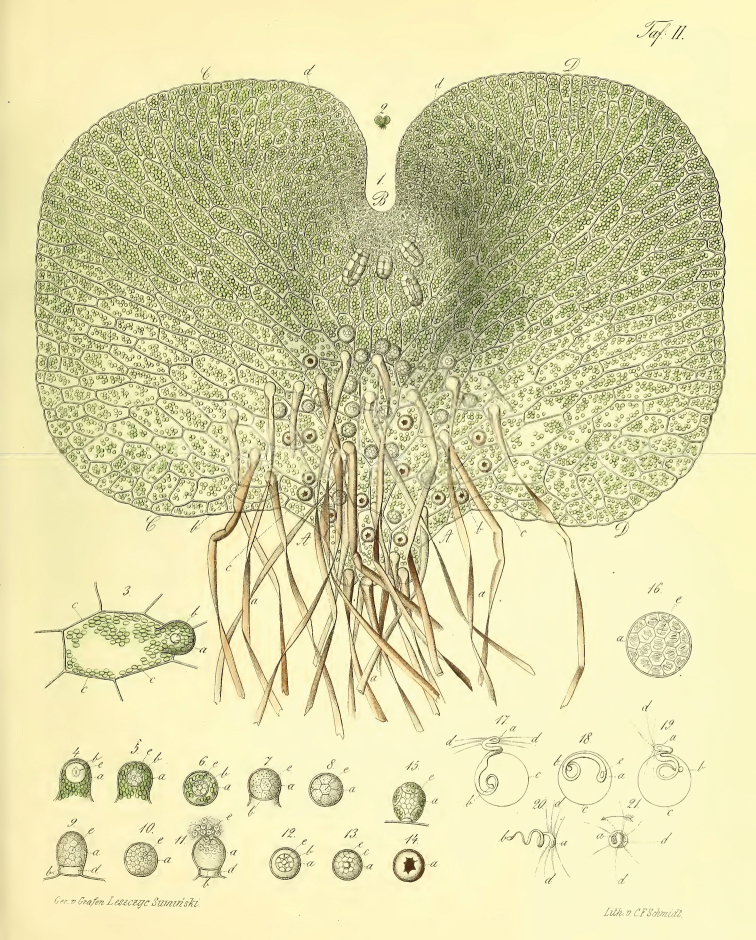
The gametophyte of fern according to Leszczic-Suminski (1848).

**Figure 3.**
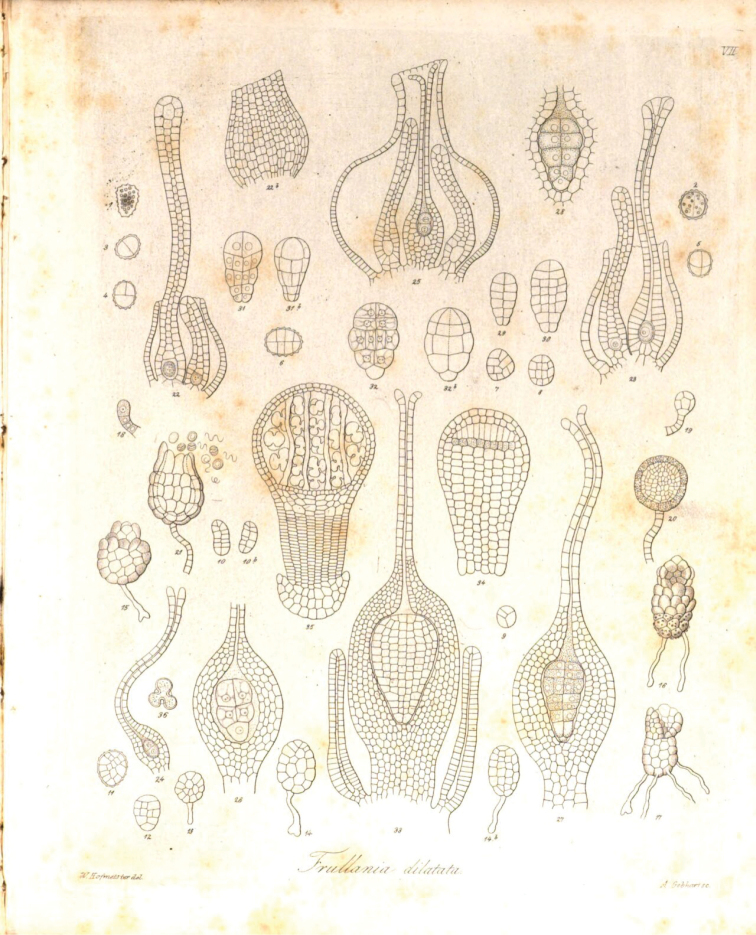
The development of liverwort according to Hofmeister (1851).

In 1848, the amateur botanist and artist M. Leszczic-Suminski (1848) was the first to understand and correctly interpret the life cycle of spore plants using the fern as an example, brilliantly illustrating the stages of this cycle with detailed color drawings (Fig. 2). Wilhelm Hofmeister, almost the same age as Suminski and also an amateur botany student without a diploma and salary, a German bookseller, immediately accepted Suminski’s discovery and studied in great detail the ontogenesis of various representatives of other groups of higher spore plants (Hofmeister 1851, 1862) (Fig. 3), generalizing it into a slender system, which to this day is the basis of evolutionary embryology of plants and botany in general. This work made it possible for the first time in the history of biology, which is more than 2000 years, to understand the fundamental difference between the life cycles of plants and the life cycles of animals. The former have two different phases in their cycle – sexual (gametophyte) and asexual (sporophyte), which successively replace each other, while animals have no such phase change. The understanding of these differences can rightly be considered one of the greatest fundamental discoveries in biology. In particular, it was only after these works that it become possible to discuss the phenomena of evolutionary embryonization and disembryonization as applied to plants. Thus, it became clear that in seed plants the gametophyte phase is completely embryonized and hidden inside the sporophyte. Here, it is appropriate to quote Hofmeister himself (Hofmeister (1862: 438): “*In more than one respect the formation of the embryo of the Coniferae is intermediate between the higher cryptogams and the phaenogams. Like the primary mother-cell of the spores of the Rhizocarpeae and Selaginellae the embryo-sac is one of the axile cells of the shoot, which in the one case becomes converted into the sporangium, in the other into the ovule. In the Coniferae also the embryo-sac soon becomes free from any mechanical connexion with the surrounding cellular tissue. The filling of the embryo-sac by the endosperm may be compared with the production of the prothallium of the Rhizocarpeae and Selaginellae*.” Moreover, Hofmeister actually constructs an evolutionary series of plants, starting from Charophyta algae and ending with spermatophyte plants: «*The phaenogams therefore form the upper terminal link of a series, the members of which are the Coniferae and Cycadeae, the vascular cryptogams, the Muscineae, and the Characeae. These members exhibit a continually more extensive and more independent vegetative existence in proportion to the gradually descending rank of the generation preceding impregnation, which generation is developed from reproductive cells cast off from the organism itself*».

Based on Hofmeister’s works, other botanists in the second half of the 19^th^ century and throughout the 20^th^ century intensively investigated numerous, previously unstudied representatives of higher and lower plants, including those from tropical regions and the southern hemisphere of the planet. Gradually, by the second half of the 20^th^ – early 21^st^ centuries, general theoretical ideas about the regularities of embryonization and disembryonization of organisms in general were formed, mainly in the works of Russian embryologists, evolutionists and theoreticians of biology. Among them are such well-known biologists as I.I. Shmalhausen, A.A. Zakhvatkin and O.M. Ivanova-Kazas and less well-known ones: A.P. Khokhryakov, E.N. Polivanova, A.L. Tikhomirova and some others (see a review of their works in Gavrilov-Zimin, 2024). Among the many names mentioned, Wilhelm Hofmeister’s name remains one of the most striking examples of a unique combination of genius, selflessness, brilliant self-education, phenomenal diligence, and unwavering pursuit of truth.
